# Supplementary material for: Architecture of the Pol III–clamp–exonuclease complex reveals key roles of the exonuclease subunit in processive DNA synthesis and repair
Source: EMBO J. 2013 Apr 2;32(9):1334–43. doi: 10.1038/emboj.2013.68 (PMC3642679; doi:10.1038/emboj.2013.68)
Supplement: Supplementary Information [file emboj201368s1.doc]

Supplementary Information

**Architecture of the Pol III-clamp-exonuclease complex reveals key roles of the exonuclease subunit in processive DNA synthesis and repair**

**Ana Toste Rêgo,^1,2^ Andrew N. Holding,^1,2^ Helen Kent,^1^ and**

**Meindert H. Lamers^1*^**

^1^ MRC laboratory of Molecular Biology, Hills Road, Cambridge, CB2 0QH, United Kingdom

^2^ These authors contributed equally to this work.

^*^ Correspondence: mlamers@mrc-lmb.cam.ac.uk


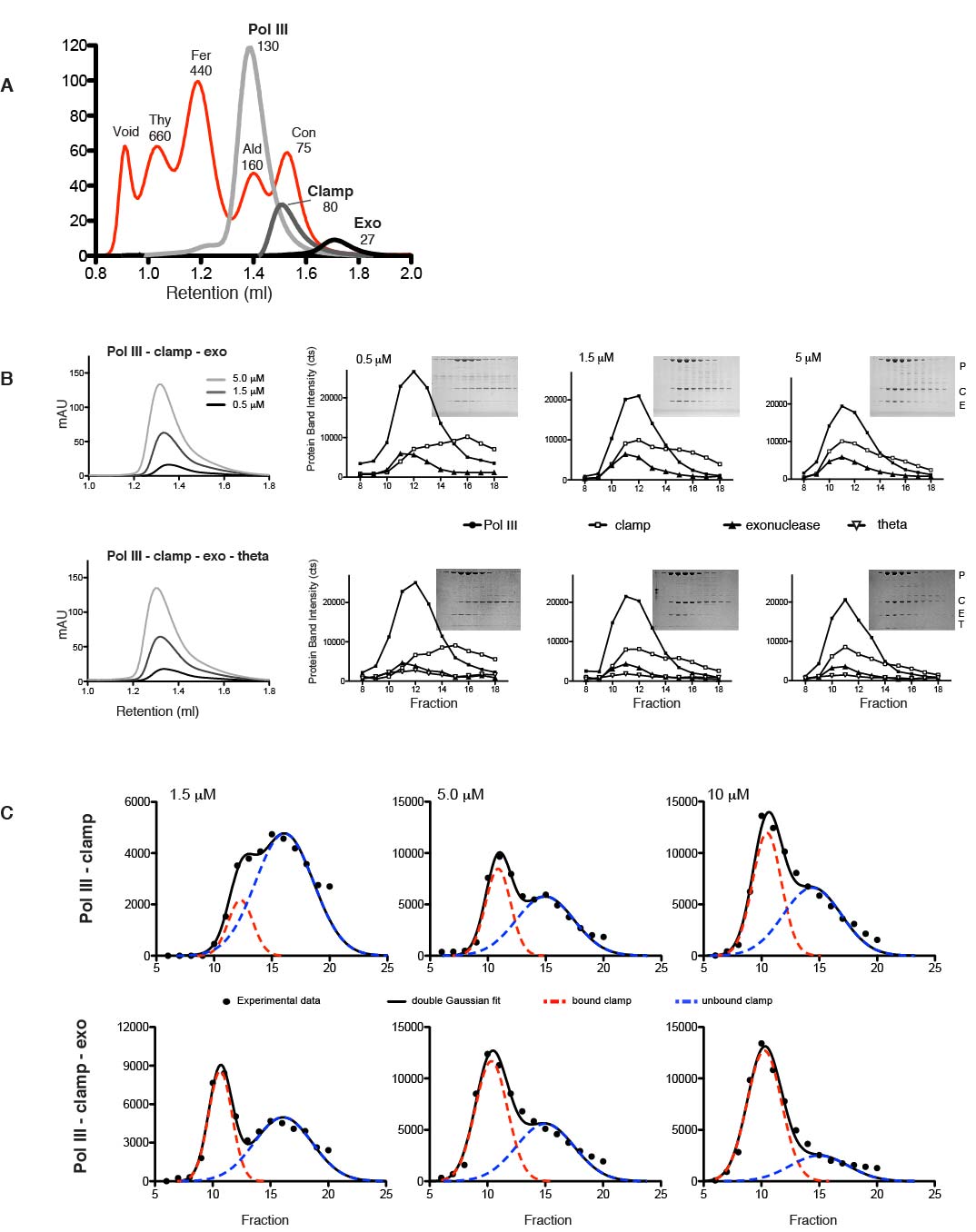


**Figure S1** (related to main Figure 1) (**A**) Chromatogram with individual runs of Pol III, clamp and exonuclease shown in grey. Molecular weight standards in red, with weights indicated in kDa. Proteins used: Thyroglobulin, Ferritin, Aldolase, Conalbumin (**B**) Addition of θ does not alter the migration pattern of the Pol III-clamp-exonuclease complex (see text for details) (**C**) Quantification of bound and unbound clamp through fitting of a ‘sum of two Gaussians’ to the band intensities of the clamp after gel filtration and SDS page shown in Figure 1.


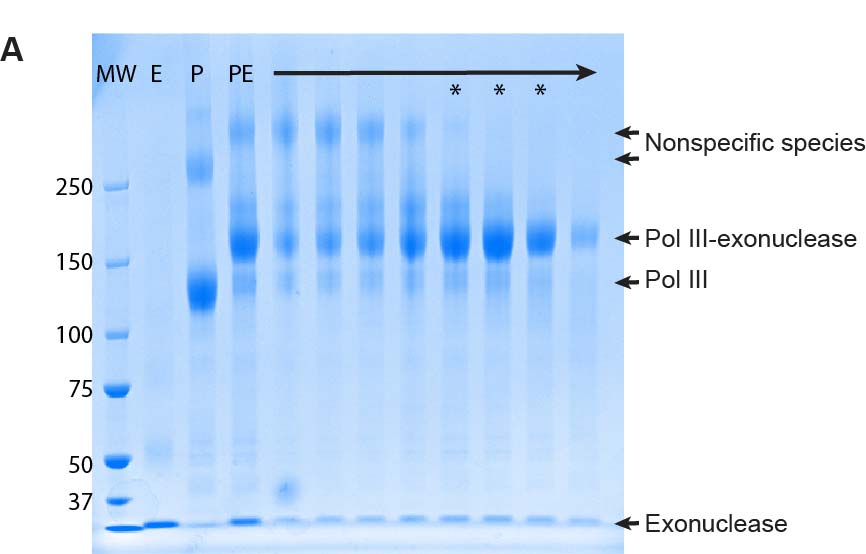


**Figure S2A** (related to main Figure 2) Purification of cross-linked products for mass spectrometry analysis. SDS-PAGE analysis of cross-linked exonuclease (lane E), cross-linked Pol III (lane P), and cross-linked Pol III-exonuclease (PE). Lane PE is also the input for the gel filtration run. Arrow indicates sequential fractions from gel filtration run, with stars indicating the fractions used for mass spec analysis. Molecular weight marker in lane MW, with weights indicated in kDa.

| **Residue 1** | **Residue 2** | **Crosslink** | **m/z** | **z** | **PPM** |
| --- | --- | --- | --- | --- | --- |
| Pol | Pol |  |  |  |  |
| 29 | 715/6 | TAPLVKK-KKPEEMAK | 464.27457 | 4+ | 0.220 |
| 29 | 714/5/6 | TAPLVKK-AMGKKKPEEMAK | 561.07312 | 4+ | 0.102 |
| 29 | 722 | TAPLVKK-KPEEMAKQR | 402.83395 | 5+ | 0.472 |
| 229 | 1009 | VAIHDGFTLDDPKRPR-VMVTKR | 533.89038 | 5+ | 0.540 |
| 316 | 595 | LKR-NGEPPLDIAAIPLDDKK | 580.075s56 | 4+ | 1.844 |
| 439 | 1009 | DAVSQIITFGTMAAKAVIR-VMVTKR | 705.89215 | 4+ | 0.219 |
| 461 | 1009 | ISKLIPPDPGMTLAK-VMVTKR | 803.79114 | 3+ | 0.784 |
| 500 | 510 | KLEGVTR-NAGKHAGGVVIAPTK | 580.08032 | 4+ | 0.363 |
| 500 | 1009 | KLEGVTR-VMVTKR | 418.99945 | 4+ | 1.111 |
| 510 | 1009 | NAGKHAGGVVIAPTK-VMVTKR | 450.45746 | 5+ | 0.538 |
| 617 | 983 | GMKDLIKR-LKDMHPTER | 437.23529 | 5+ | 0.115 |
| 621 | 983 | DLIKR-LKDMHPTER | 622.66815 | 3+ | 0.149 |
| 855 | 872 | NKGGYFR-TDTKK | 393.46091 | 4+ | 0.362 |
| 983 | 922 | LKDMHPTER-GKVITAAGLVVAAR | 637.60626 | 4+ | 1.688 |
| Exo | Pol |  |  |  |  |
| 120 | 1009 | DIPKTNTFCK-VMVTKR | 513.77069 | 4+ | 0.346 |
| 136 | 229 | KMFPGKR-VAIHDGFTLDDPKRPR | 473.75958 | 6+ | 1.120 |
| 136 | 510 | KMFPGKR-NAGKHAGGVVIAPTK | 397.22406 | 6+ | 1.901 |
| 136 | 1009 | KMFPGKR-VMVTKR | 434.2533 | 4+ | 0.195 |
| 141 | 229 | KMFPGKR-VAIHDGFTLDDPKRPR | 559.90088 | 5+ | 0.726 |
| 141 | 1009 | MFPGKR-VMVTKR | 391.71753 | 4+ | 0.965 |
| 158 | 1009 | YEIDNSKR-VMVTKR | 474.50644 | 4+ | 0.833 |
| 235 | 29 | KGGSCLWRA-TAPLVKK | 454.50378 | 4+ | 0.683 |
| 235 | 714 | KGGSCLWR-AMGKK | 545.62134 | 3+ | 0.642 |
| Clamp | Pol |  |  |  |  |
| 277 | 872 | AAILSNEKFR-TDTKK | 612.66669 | 3+ | 0.337 |
| 277 | 1009 | AAILSNEKFR-VMVTKR | 659.70227 | 3+ | 1.376 |
| Clamp | Exo |  |  |  |  |
| 277 | 136 | AAILSNEKFRGVR-KMFPGKR | 493.08282 | 5+ | 0.410 |
| 277 | 141 | AAILSNEKFR-KMFPGKR | 538.05371 | 4+ | 0.935 |

**Figure S2B** (related to main Figure 2) Summary of cross-linked peptides with precursor mass error (PPM) for each cross-linked position. Additionally, we have provided the precursor mass:charge ratio (m/z), and charge (z). For clarity, where multiple version of the same cross-links were detected only one sequence has been provided. No additional modifications where present on the peptides listed.

**
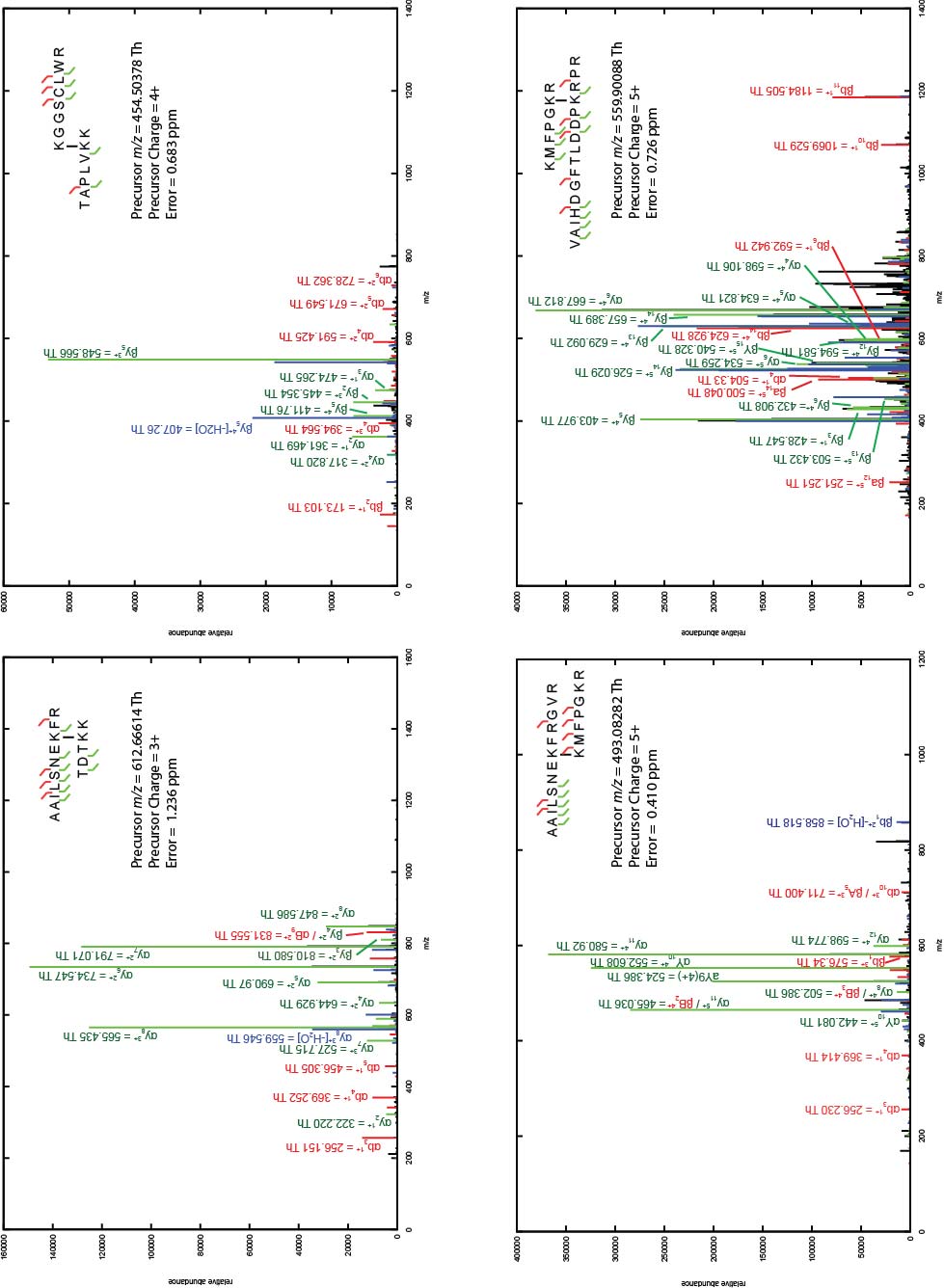
**

**Figure S2C** (related to main Figure 2) Fragmentation spectra from four different cross-linked peptides. Peptides were scored probabilistically by matching fragmentation ions using an algorithm adapted from previously described linear peptide analysis scoring techniques (Cox et al, J. Proteome Res. 10: 1794-1805, Holding et al. 2013, manuscript in preparation). A minimum score of 250 was used as a cut-off in identifying peptides. This figure was calculated as -10 x Log_e_ (the probability that the identified number of theoretical ions or more was matched to the fragmentation spectra by chance).

**
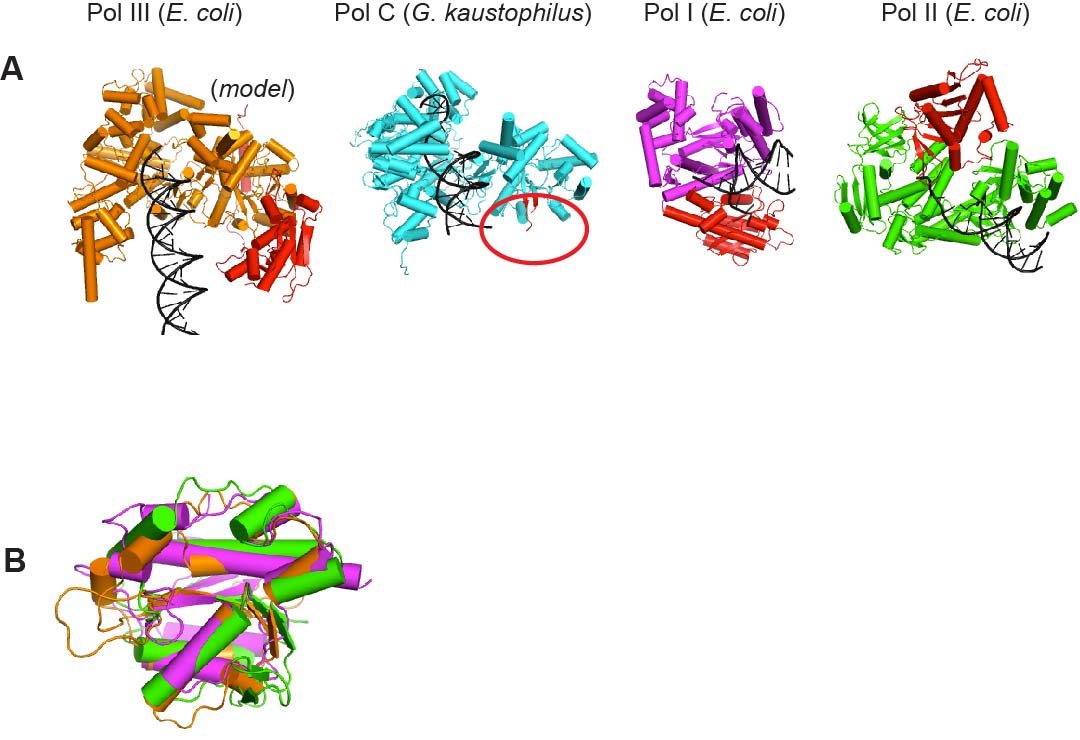
**

**Figure S3** (related to main Figure 2) Position of the exonuclease in different bacterial DNA polymerases. (**A**) Model of Pol III bound to exonuclease and DNA, and crystal structures of Pol C, Pol I and Pol II, aligned using the thumb, palm and fingers domain of each polymerase. Exonucleases are coloured in red, with deleted exonuclease in PolC indicated with red circle. (**B**) Overlay of the exonucleases from Pol I (magenta), Pol II (green), and Pol III (orange)


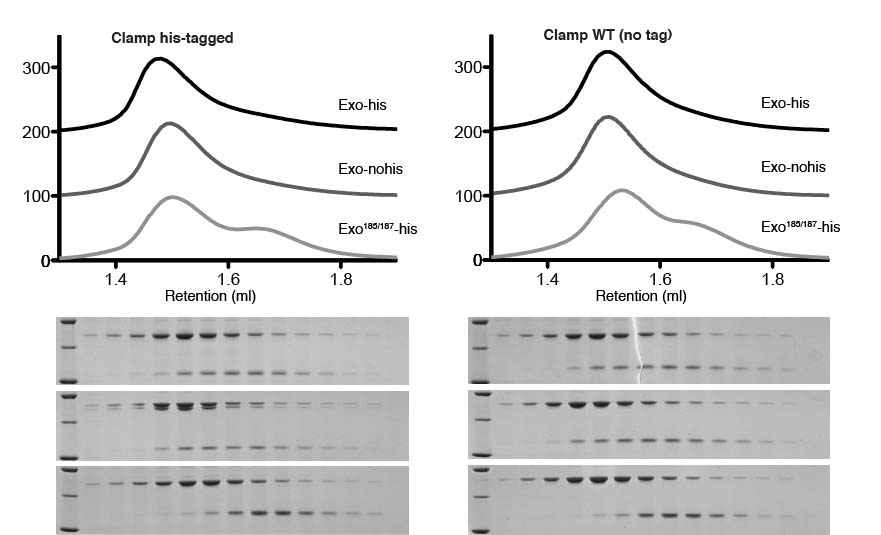


**Figure S4** (related to main Figure 3) Verification that the his-tag on the proteins does not affect the interaction. His-tagged clamp (left panels) or non-tagged clamp (right panels) was incubated with either: his-tagged exonuclease (top), non-tagged exonuclease (middle), or his-tagged mutant exonuclease^185/187^ (bottom). The presence of a his-tag on either clamp or exonuclease has no effect on the interaction between the two proteins. In contrast, mutation of the canonical clamp binding motif in the exonuclease disrupts the interaction with the clamp.


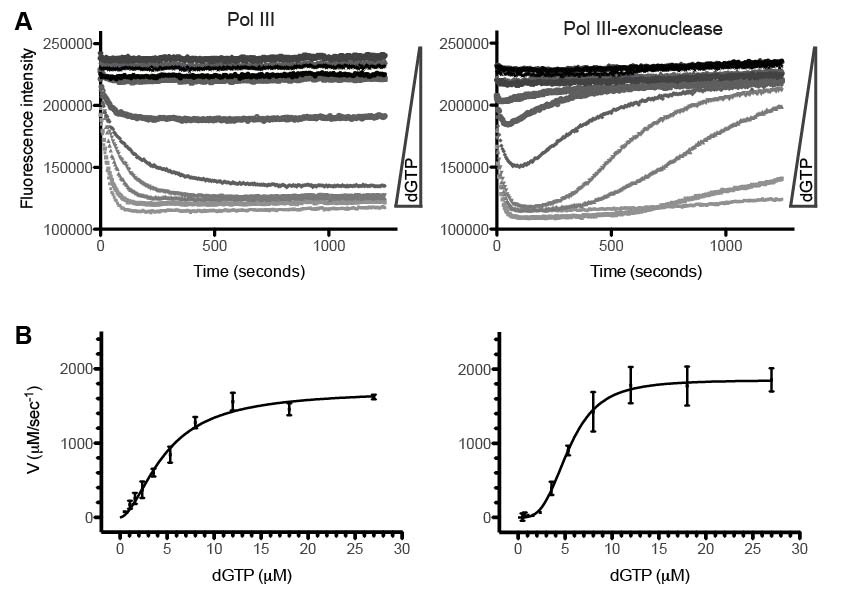


**Figure S5** (related to main Figure 4) Two examples of real-time primer extension assays (**A**) Activities of Pol III and Pol III-exonuclease at increasing dGTP concentrations. Note that at medium dGTP concentration the exonuclease rapidly takes over from the polymerase and restores fluorescence intensity to starting values. At high concentrations the activity of the exonuclease only becomes apparent after >500 seconds due to the excess of dGTP. (**B**) Determination of *V*_max_ by using the initial linear velocity (~ first 30 seconds) of the graphs shown in A. Experiments were repeated three times.


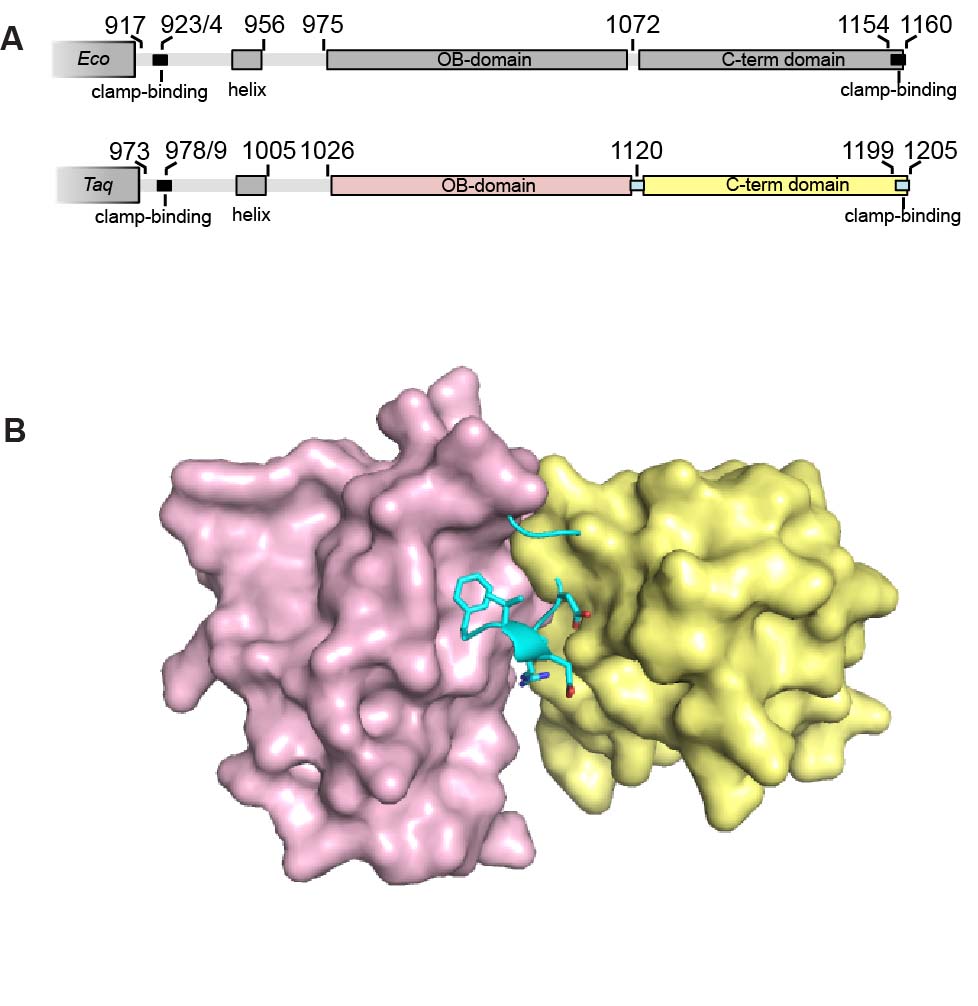


**Figure S6A and B** (related to main Figure 5) The C-terminal clamp binding of Pol III is wedged between two domains. (**A**) Schematic view of the C-terminal tail of Pol III from *E. coli* (top) and *T. aquaticus* (bottom). The oligonucleotide/ oligosaccharide binding (OB) domain and C-terminal domain are colored in pink and yellow respectively. (**B**) Structure of the two C-terminal domain of Taq Pol III (2HPI.pdb). Linker between the two domains indicated with light blue coil. C-terminal clamp binding motif is indicated in light blue cartoon with side chains indicated in stick model.

**
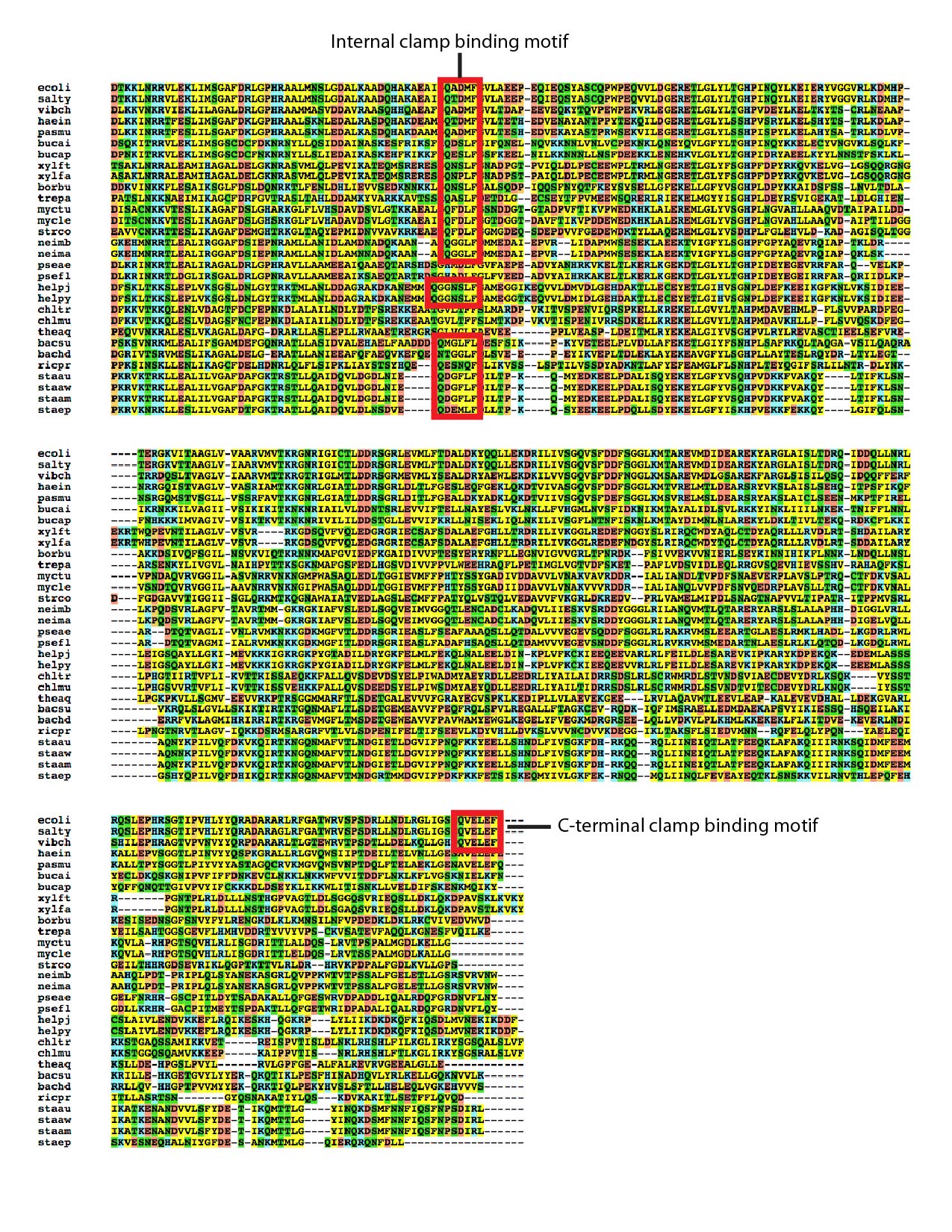
­­­­**

**Figure S6C** (related to main Figure 5) The C-terminal clamp binding motif is not conserved in Pol III homologs. Alignment of the C-terminal region of Pol III from 30 different bacterial species. The internal clamp binding motif is found in 24 out of 30 sequences, while the C-terminal binding motif is only found in 3 sequences. Clamp binding motifs are indicated in red boxes. Residue coloring: red: negatively charged, blue, positively charged, green: polar uncharged, yellow: hydrophobic.

**
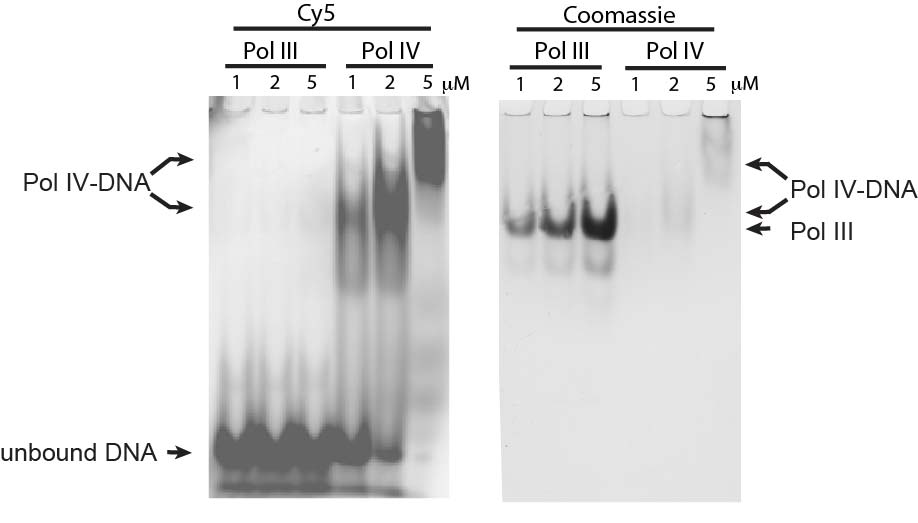
**

**Figure S7** (related to main Figure 8) Pol III has a very weak affinity for DNA.

Gel mobility shift of a primed DNA substrate by Pol III and Pol IV. 1 μM DNA (Cy5-labelled, 25 base pairs double stranded DNA with a 5 nucleotide single stranded overhang) was incubated with 1, 2, or 5 μM protein for 30 minutes on ice, and then separated on a native 6% acrylamide gel in TBE buffer. While Pol III has no affinity for the DNA under these conditions, Pol IV readily shifts the DNA. The higher band that is visible in the 5μM Pol IV lane may be explained by two Pol IV molecules binding to one DNA molecule. Cy5-labeled DNA was imaged with a Typhoon scanner (GE Healthcare). Gels were afterwards stained with Coomassie Brilliant blue to visualize the proteins.
